# Prevalence and correlates of muscle dysmorphia in a sample of boys and men in Canada and the United States

**DOI:** 10.1186/s40337-025-01233-x

**Published:** 2025-03-17

**Authors:** Kyle T. Ganson, Deborah Mitchison, Rachel F. Rodgers, Stuart B. Murray, Alexander Testa, Jason M. Nagata

**Affiliations:** 1 Factor-Inwentash Faculty of Social Work, University of Toronto, Toronto, ON Canada; 2 Discipline of Clinical Psychology, Graduate School of Health, Faculty of Health, University of Technology Sydney, Sydney, NSW Australia; 3 Eating Disorder and Body Image Network, Translational Health Research Institute, Western Sydney University, Campbelltown, NSW Australia; 4 Department of Applied Psychology, Northeastern University, Boston, MA USA; 5 Department of Psychiatric Emergency & Acute Care, Lapeyronie Hospital, Montpellier, France; 6 Centre de Recherche et d’Innovation Clinique, Hôpital du Cotentin, Cherbourg, France; 7 Department of Psychiatry and the Behavioral Sciences, University of Southern California, Los Angeles, CA USA; 8 Department of Management, Policy and Community Health, University of Texas Health Science Center at Houston, Houston, TX USA; 9 Department of Pediatrics, University of California, San Francisco, San Francisco, CA USA

**Keywords:** Muscle dysmorphia, Canada, United States, Gender, Boys, Men, Transgender men, Epidemiology, Mental health

## Abstract

**Background:**

Muscle dysmorphia is a significant mental health condition that has been under-researched in epidemiological, community-based studies. Therefore, this study aimed to identify the prevalence and correlates of probable muscle dysmorphia among a sample of Canadian (*n* = 784) and American (*n* = 563) boys and men ages 15–35 years.

**Methods:**

The sample comprised 1,488 boys and men who completed a variety of measures and items to capture sociodemographic characteristics and muscle dysmorphia symptoms. Diagnostic criteria were applied to identify probable muscle dysmorphia among the sample. Unadjusted (e.g., chi-square tests, independent samples *t-*tests) and adjusted (e.g., logistic regression) analyses were used to determine the sociodemographic factors (e.g., age, body mass index, gender, race/ethnicity, sexual orientation, education, relationship status, and country) associated with cases of probable muscle dysmorphia.

**Results:**

The prevalence of probable muscle dysmorphia was 2.8% (95% confidence interval 2.0-3.7%). Aside from lower body mass index among those with probable muscle dysmorphia, there were no significant demographic differences between those with and without probable muscle dysmorphia across ages, genders, races/ethnicities, and sexual orientations. Those with probable muscle dysmorphia had significantly higher scores on standardized measures of muscle dysmorphia symptomatology and muscularity-oriented attitudes and behaviors compared to those without probable muscle dysmorphia.

**Conclusions:**

Findings underscore that muscle dysmorphia may be more prevalent among boys and men in Canada and the United States than previously thought, highlighting the need for more research, prevention, assessment, and intervention efforts. The minimal differences across sociodemographic factors are notable, highlighting the need for an inclusive understanding of muscle dysmorphia.

**Supplementary Information:**

The online version contains supplementary material available at 10.1186/s40337-025-01233-x.

## Abstract (plain language summary)

Probable muscle dysmorphia occurred at a prevalence of 2.8% among a sample of boys and men in Canada and the United States. There were minimal sociodemographic differences between those with and without probable muscle dysmorphia. The findings from this study underscore that muscle dysmorphia may be more common among boys and men than previously thought and that the condition occurs across sociodemographic groups. Prevention and intervention efforts for public health and healthcare professionals should consider the broad representation of muscle dysmorphia among boys and men.

**Supplementary Information:**

The online version contains supplementary material available at 10.1186/s40337-025-01233-x.

## Introduction

Muscle dysmorphia, a specifier of body dysmorphic disorder, is a mental health condition that is described as the pathological pursuit of muscularity [1, 2]. Symptoms of muscle dysmorphia include a preoccupation with a perceived lack of muscularity and obsessive and compulsive behaviors aimed at increasing muscularity (e.g., excessive weight training, strict dietary practices) [1–3]. The onset of muscle dysmorphia most commonly occurs during the transition from adolescence to young adulthood [4], where significant biological, psychological, and social changes occur [5], and body image concerns are ubiquitous [6]. Additionally, given the pressures to pursue a muscular body for boys and men [7], muscle dysmorphia more commonly impacts this gender group [3, 8].

The current research on muscle dysmorphia faces two major challenges insofar as it aids an understanding of the epidemiology of this condition. First, a majority of prior research has primarily focused on smaller samples of individuals who fall into “high-risk” groups, such as bodybuilders [9–12]. This narrow focus results in significant unknowns about how muscle dysmorphia may present in the broader population, including the prevalence and burden of the condition, as well as risk and protective factors [13]. Recently, however, a growing body of research has begun to investigate muscle dysmorphia among larger community- and population-based samples. In Canada, a recent study of over 2,200 adolescents and young adults ages 16–30 years across all 13 provinces and territories found that 26% of boys and men in the study were at clinical risk for muscle dysmorphia, defined as scoring 40 or above on the Muscle Dysmorphic Disorder Inventory (MDDI) [14]. In another study conducted among a representative sample of over 3,600 high school students ages 11–19 years in Australia, 2.2% of adolescent boys met research-based diagnostic criteria for muscle dysmorphia [13]. Similarly, among a sample of Spanish undergraduate men, 1.3% met the criteria for muscle dysmorphia via a clinical interview [15]. These three studies highlight the relatively high prevalence of clinically relevant muscle dysmorphia symptomatology in adolescents and young adults, particularly boys and men, and set a clear impetus for further research to corroborate these findings and further investigate muscle dysmorphia phenomenology within the broader population.

A second challenge is that much of the prior research has measured muscle dysmorphia symptomatology, using measures such as the MDDI, therefore, describing symptoms on a continuum versus investigating clinically relevant symptoms. Consistent across studies, muscle dysmorphia symptomatology, most notably drive for muscularity, tends to be higher among cisgender and transgender men compared to cisgender women, transgender women, and gender-expansive people [14, 16–18]. However, studies conflating an elevated drive for muscularity with clinical presentations of muscle dysmorphia run the risk of (1) inflating the prevalence rates of muscle dysmorphia, particularly given the ubiquity of a heightened drive for muscularity, and (2) under-detecting the severity and disease burden associated with its course. While research examining muscle dysmorphia symptomatology on a continuum is helpful in aiding an understanding of broader societal norms, and at what level of frequency symptoms become problematic, it is also important to examine cohorts who meet agreed-upon diagnostic criteria to establish the frequency, distribution, and cost of conditions at a population-level. However, the empirical literature lacks research implementing diagnostic criteria and recruitment of clinical samples [19]. Therefore, more data from individuals with muscle dysmorphia based on clinical criteria, particularly in epidemiological studies, are needed to understand the presentation of the condition to inform assessment, diagnosis, and treatment of the condition [20]. While clinician interview remains the gold standard in detecting and diagnosing muscle dysmorphia, recent efforts have successfully developed bespoke self-report survey items, which are based on DSM-5 criteria, that have been used to estimate prevalence in large-scale epidemiological studies [13] and require replication studies to increase validity.

Given the multiple ways in which prior research on muscle dysmorphia is limited, the current study had three specific aims. First, this study aimed to describe the prevalence of muscle dysmorphia among a community-based sample of boys and men in Canada and the United States. Second, this study aimed to identify the sociodemographic correlates of muscle dysmorphia. Finally, this study aimed to compare levels of muscle dysmorphia symptomatology and muscularity-oriented attitudes and behaviors based on standardized measures among those with and without muscle dysmorphia.

## Methods

The Study of Boys and Men was an online survey on the contemporary lives and experiences of boys and men in Canada and the United States. Participants were recruited via Instagram and Snapchat advertisements during March and April 2024. Social media advertisements targeted males (sex), ages 15–35, and Canada and the United States (countries). No other targeting criteria were used. Advertisements were generic in nature to recruit a broad range of participants (i.e., “Don’t miss your chance to make your voice heard! Participate today in: The Study of Boys and Men, an international research study of the contemporary lives of boys and men.”). The online survey was conducted via Qualtrics. Several recommended features were used to protect against bot infiltration [21], including reCAPTCHA verification, attention checks, and honeypot items. Additionally, several Qualtrics features were utilized, including “prevent multiple submissions” (i.e., preventing participants from taking the survey multiple times) and “block indexing options” (i.e., blocking search engines from including the survey in their search results). Responses were also monitored daily to ensure there was not a significant and unusual increase in responses, and responses were assessed for straight lining, unanswered questions, and illogical open responses.

A total of 1,791 individuals completed the online survey, of which 1 response was removed for a wrong answer on an attention check, 11 responses were removed for not completing both attention checks, 4 responses were removed for having greater than 50% unanswered questions, 6 responses were removed for completing the survey in less than 10 min, 212 responses were removed for failing reCAPTCHA verification, and 4 duplicate responses were removed. This resulted in a sample of 1,553 responses. This analysis only included those who had no missing data for the probable muscle dysmorphia variable, resulting in a final analytic sample of *N* = 1,488. Participants were able to enter a drawing to win one of three Apple iPads or one of 25 $25 Starbucks gift cards as compensation for completing the survey. Online advertisements did not reference compensation for survey participation. All participants provided informed consent via the survey checkbox. Of note, all participants were provided a list of Canadian and American resources that provide information and support for people with eating disorders, body image concerns, and overall mental health needs. Ethics approval for the study was received from the Health Sciences Research Ethics Board at the University of Toronto (#45880).

### Measures

#### Muscle dysmorphia

A probable muscle dysmorphia diagnosis was assessed based on the operationalization outlined by Mitchison et al. (2021) that mirrored the original muscle dysmorphia diagnostic criteria [22]. These criteria incorporate various empirically supported measures and two researcher-developed items to evaluate preoccupation with muscularity and the drive for muscularity (Criterion A), functional impairment and clinically significant distress (Criterion B), and the absence of an eating disorder (Criterion C) [1, 22]. Empirically supported measures included the Muscle Dysmorphic Disorder Inventory [23], the Drive for Muscularity Scale [24], the Eating Disorder Examination Questionnaire 6.0 [25], the Pediatric Quality of Life Inventory (adolescent and young adult versions) [26, 27], and the Kessler Psychological Distress Scale [28]. Items developed by the research team focused on adherence to a high-protein diet and the use of anabolic-androgenic steroids. See Supplementary Table 1 for full muscle dysmorphia criteria, including the internal consistency of the measures (ranging from good to excellent) included in the operationalization. Note that participants in this study were not formally diagnosed with muscle dysmorphia via a clinical interview. Therefore, we refer to these participants as experiencing “probable muscle dysmorphia.”

#### Muscle dysmorphic disorder inventory

The Muscle Dysmorphic Disorder Inventory (MDDI) is a 13-item measure that assesses symptoms of muscle dysmorphia [23]. The MDDI uses a 5-point scale (1 = *never*; 5 = *always*) and scores are calculated by summing the 13 scale items, with a higher score indicating greater symptom severity of muscle dysmorphia. The MDDI encompasses three subscales. The Drive for Size subscale measures a desire to increase muscular bulk (e.g., “I wish I could get bigger.”), the Appearance Intolerance subscale measures both dissatisfaction and avoidance associated with appearance (e.g., “I feel like I have too much body fat.”), and the Functional Impairment subscale measures the impairment associated with muscle dysmorphia symptomatology, such as excessive exercising (e.g., “I feel depressed when I miss one or more workout days.”) [23]. Internal reliability using Cronbach’s alpha was 0.80 for the MDDI total score, 0.86 for the Drive for Size subscale, 0.82 for the Appearance Intolerance subscale, and 0.85 for the Functional Impairment subscale among the sample. A score of ≥ 40 on the MDDI was used to determine clinical risk for muscle dysmorphia [14, 18, 29, 30].

#### Drive for muscularity

The Drive for Muscularity Scale (DMS) is a 15-item measure that assesses muscularity-oriented attitudes and behaviors [24]. Sample items include, “I wish that I were more muscular” and “I think I would feel more confident if I had more muscle mass”. Each item is scored on a 6-point scale (1 = *never*; 6 = *always*). Scores are summed to create a total score, with higher scores indicating greater drive for muscularity [24]. Internal reliability using Cronbach’s alpha was 0.91.

#### Sociodemographic variables

Sociodemographic variables included self-reported age, race/ethnicity (White, Black, Asian, Latin American, multi-racial, and other race/ethnicity), sexual orientation (heterosexual, gay, bisexual, queer, questioning, and other sexual orientation), highest completed education (high school diploma or less, college or undergraduate degree, master’s degree or higher, and other education), relationship status (single, in a relationship), and country of residence (based on self-reported postal codes [Canada] and zip codes [United States]). Gender was assessed using self-reported sex assigned at birth and current gender identity (cisgender boy/man, transgender boy/man, gender expansive, and other gender). See Supplementary Table 2 for full demographic questions. Self-reported height and weight were used to calculate body mass index (BMI; kilograms/meters^2^).

### Statistical analysis

Descriptive statistics were used to calculate the means, standard deviations, and frequencies of the variables under study, including the prevalence of probable muscle dysmorphia (Aim 1). Chi-square tests and independent samples *t*-tests were used to assess the sociodemographic differences between those with and without probable muscle dysmorphia (Aim 2). One logistic regression analysis was used to assess the associations between the sociodemographic factors (e.g., age, BMI, gender, race/ethnicity, sexual orientation, education, relationship status, and country) and probable muscle dysmorphia (Aim 2). Welch’s *t*-tests were used given unequal variances to assess the mean score differences on the MDDI and DMS between those with and without probable muscle dysmorphia (Aim 3). Finally, one chi-square test was used to determine the differences between those with and without probable muscle dysmorphia on scoring ≥ 40 on the MDDI (Aim 3). Listwise deletion was used to account for missing data given that there was minimal missing data (average of < 5% across variables) and because the sample size is relatively large, raising minimal issues with statistical power [31, 32]. See Supplementary Table 3 for patterns of missing data. To determine the minimum sample size required to test the study hypotheses, power analyses were conducted using G*Power [33]. Results indicated that a minimum sample of size 1,484 achieves 80% power for detecting an odds ratio of 1.2 at a significance level of α = 0.05 for a logistic regression analysis with eight predictor variables. For comparisons (*t-*test) between those with and without probable muscle dysmorphia, a minimum total sample of 1,438 is required to achieve 80% power for detecting a medium effect (0.45), at a significance level of α = 0.05. Statistical significance was determined using *p* <.05. All statistical analyses were conducted using StataMP 18.

## Results

The mean age of the sample was 24.1 (SD = 5.6) years old (range: 15–35 years) and 82.4% identified as cisgender boys or men, 65.7% as White, and 48.2% as heterosexual (Table 1). Nearly half (43.8%) reported completing a high school diploma or less education and 54.8% reported being single. Slightly more than half (58.4%) were from Canada. Among the sample, 2.8% (95% confidence interval [CI] 2.0-3.7%, *n* = 41) met the criteria for probable muscle dysmorphia.

**Table 1:** Sociodemographic characteristics and probable muscle dysmorphia comparisons among a sample of boys and men (*N* = 1,488)

	Overall *N* = 1,488	No MD *n* = 1,447	MD *n* = 41		
	% (*n*)	% (*n*)	% (*n*)	*p* ^a^	*t / V* ^b^
Age (*M* [SD])	24.1 (5.6)	24.1 (5.6)	22.9 (5.7)	0.163	1.39
Age Groups				0.146	-0.04
< 18 years	12.2 (181)	12.0 (19.5)	19.5 (8)		
≥ 18 years	87.81 (1,304)	88.0 (1,271)	80.6 (33)		
BMI (*M* [SD])	25.2 (6.2)	25.3 (6.2)	22.1 (3.8)	**0.001**	**3.34**
Gender				0.064	0.06
Cisgender Boy/Man	82.4 (1,199)	82.0 (1,160)	95.1 (39)		
Trans Boy/Man	7.4 (108)	7.5 (106)	4.9 (2)		
Gender Expansive and Other	10.2 (148)	10.5 (148)	0.0 (0)		
Race/Ethnicity				0.206	0.07
White	65.3 (922)	65.3 (895)	65.9 (27)		
Black	3.1 (43)	2.6 (39)	9.8 (4)		
Asian	11.0 (155)	11.1 (152)	7.3 (3)		
Latin American	4.0 (57)	4.1 (56)	2.4 (1)		
Other	3.6 (51)	3.6 (50)	2.4 (1)		
Multi-Racial	13.0 (183)	13.0 (178)	12.2 (5)		
Sexual Orientation				0.960	0.02
Heterosexual	48.2 (678)	48.2 (659)	46.3 (19)		
Gay	19.9 (280)	19.8 (271)	22.0 (9)		
Bisexual	13.9 (196)	13.9 (190)	14.6 (6)		
Queer	8.0 (112)	7.9 (108)	9.8 (4)		
Questioning and Other	10.1 (142)	10.2 (139)	7.3 (3)		
Education				0.059	0.06
High School Diploma or Less	43.8 (620)	43.4 (596)	58.5 (24)		
College or Undergraduate Degree	39.6 (561)	39.7 (546)	36.6 (15)		
Master’s Degree or Higher	16.5 (234)	16.9 (232)	4.9 (2)		
Relationship Status				**0.038**	**-0.06**
Single	54.8 (780)	54.3 (751)	70.7 (29)		
In a Relationship	45.2 (643)	45.7 (631)	29.3 (12)		
Country				0.379	0.02
Canada	58.4 (759)	58.6 (740)	51.4 (19)		
United States	41.6 (541)	41.4 (523)	48.6 (18)		
Probable Muscle Dysmorphia	2.8 (41)	-	-	-	

^a^ Statistical significance was determined using chi-square tests for categorial variables and independent samples *t*-tests for continuous variables

^b^*t* statistic from independent samples *t*-tests for continuous variables and Cramér’s V for categorical variables

**Boldface** indicates statistical significance at *p* <.05.

MD = Muscle dysmorphia; *M* = Mean; SD = Standard deviation; BMI = Body mass index

There were few statistically significant sociodemographic differences, except for BMI, between those with and without probable muscle dysmorphia in both unadjusted (Table 1) and adjusted (i.e., logistic regression model adjusting for age, BMI, gender, race/ethnicity, sexual orientation, education, relationship status, and country; Table 2) analyses. For parsimony, only adjusted analyses are reported. Lower BMI was significantly associated with probable muscle dysmorphia (adjusted odds ratio [AOR] 0.85, 95% CI 0.77–0.93), and having a master’s degree or higher, compared to a high school diploma or less, was significantly associated with lower odds of probable muscle dysmorphia (AOR 0.09, 95% CI 0.01–0.77).

**Table 2:** Sociodemographic associations with probable muscle dysmorphia among a sample of boys and men (*N* = 1,553)

	AOR (95% CI)	*p*
Age	1.04 (0.95–1.12)	0.390
BMI	**0.85 (0.77–0.93)**	**0.001**
Gender		
Cisgender Boy/Man	Ref.	Ref.
Trans Boy/Man	0.36 (0.08–1.71)	0.200
Gender Expansive and Other	-	-
Race/Ethnicity		
White	Ref.	Ref.
Black	2.62 (0.69–9.88)	0.155
Asian	0.89 (0.25–3.12)	0.857
Latin American	0.65 (0.08–5.11)	0.687
Other	1.22 (0.15–10.07)	0.848
Multi-Racial	1.01 (0.37–2.78)	0.976
Sexual Orientation		
Heterosexual	Ref.	Ref.
Gay	1.66 (0.69-4.00)	0.257
Bisexual	1.68 (0.62–4.57)	0.308
Queer	3.07 (0.89–10.62)	0.076
Questioning and Other	1.02 (0.28–3.75)	0.975
Education		
High School Diploma or Less	Ref.	Ref.
College or Undergraduate Degree	0.76 (0.33–1.75)	0.527
Master’s Degree or Higher	**0.09 (0.01–0.77)**	**0.028**
Other	-	-
Relationship Status		
Single	Ref.	Ref.
In a Relationship	0.68 (0.32–1.45)	0.320
Country		
Canada	Ref.	Ref.
United States	1.07 (0.53–2.15)	0.852

Note: Outputs refer to a single logistic regression analysis with age, BMI, gender, race/ethnicity, sexual orientation, education, relationship status, and country included as the independent variables and probable muscle dysmorphia as the dependent variable

**Boldface** indicates statistical significance at *p* <.05.

AOR = Adjusted odds ratio; CI = Confidence interval; BMI = Body mass index; Ref. = Reference group

**Fig. 1:**
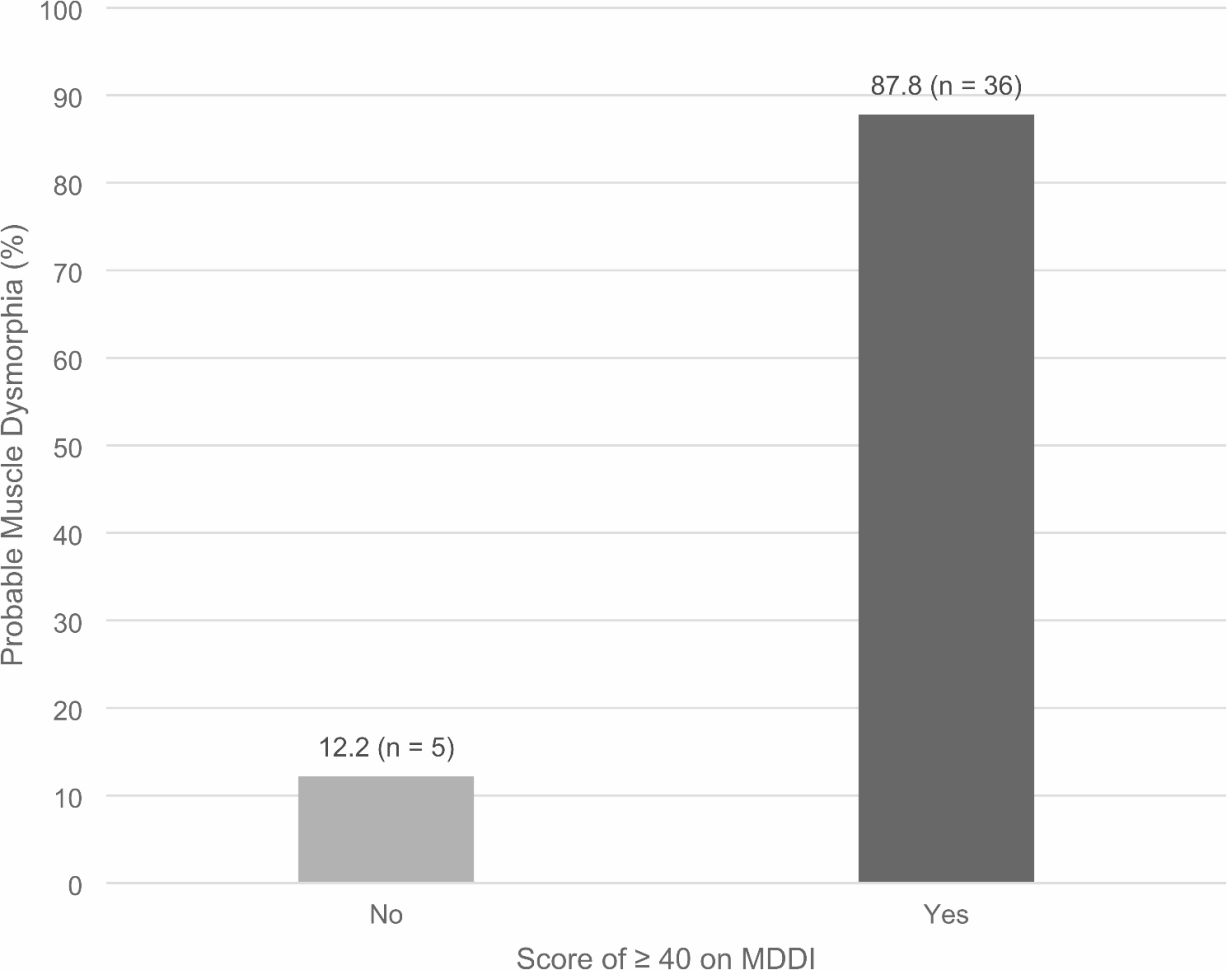
Probable muscle dysmorphia by a score of ≥ 40 on the MDDI. Statistical significance determined using chi-square test. * *p* <.001. MDDI = Muscle Dysmorphic Disorder Inventory. Note: Scores < 40 on the MDDI included 36 (*n* = 1), 37 (*n* = 1), 38 (*n* = 2), and 39 (*n* = 1)

There were no significant differences between those with and without probable muscle dysmorphia on the MDDI Appearance Intolerance scores (Table 3). However, those with probable muscle dysmorphia had higher MDDI total and DMS scores. Specifically, those with probable muscle dysmorphia had higher Drive for Size scores (*M* = 22.5, SD = 1.4), Functional Impairment scores (*M* = 10.8, SD = 4.5), MDDI Total scores (*M* = 45.3, SD = 5.5), and total DMS scores (*M* = 65.8, SD = 10.7) compared to those without muscle dysmorphia. Finally, a strong majority of participants with probable muscle dysmorphia scored ≥ 40 on the MDDI (87.8%, *n* = 36), indicating clinical risk, compared to 2.2% (*n* = 5) who scored < 40 on the MDDI (*p* <.001; Fig. 1).

**Table 3:** Unadjusted comparisons between probable muscle dysmorphia and MDDI and DMS scores

	No MD *n* = 1,447	MD *n* = 41	*p* ^a^	d	t
	*M* (SD)	*M* (SD)			
MDDI Scores					
Drive for Size	**12.5 (4.9)**	**22.5 (1.4)**	**< 0.001**	**2.8**	**-38.9**
Appearance Intolerance	11.6 (4.5)	12.0 (3.1)	0.533	0.1	-0.9
Functional Impairment	**7.3 (3.6)**	**10.8 (4.5)**	**< 0.001**	**0.9**	**-5.0**
Total	**31.4 (8.7)**	**45.3 (5.5)**	**< 0.001**	**1.9**	**-15.6**
DMS Score	**43.0 (14.5)**	**65.8 (10.7)**	**< 0.001**	**1.8**	**-13.3**

^a^ Statistical significance was determined using Welch’s *t-*tests given unequal variances

**Boldface** indicates statistical significance at *p* <.05.

MD = Muscle dysmorphia; *M* = Mean; SD = Standard deviation; MDDI = Muscle Dysmorphic Disorder Inventory; DMS = Drive for Muscularity Scale

## Discussion

Muscle dysmorphia is a serious mental health condition, however, the vast majority of prior research, until recently, has neglected to utilize diagnostic criteria [19]. Utilizing operationalized proposed diagnostic criteria for muscle dysmorphia [13, 22], findings relevant to our first aim were that the prevalence of probable muscle dysmorphia among nearly 1,500 boys and men in Canada and the United States was 2.8%. Importantly, the boys and men in this sample included representation across sociodemographic identifiers, specifically related to gender, race/ethnicity, and sexual orientation. This prevalence is similar to the 2.2% prevalence among boys documented in the original study that used the same operationalization to establish the prevalence of probable muscle dysmorphia among high school-aged adolescents in Australia [13]. However, the prevalence is higher than a sample of Spanish undergraduate men (1.3%) who were assessed via a clinical interview [15]. Indeed, greater healthcare and public health attention to muscle dysmorphia is needed to continue to understand the scope of the condition, as well as develop evidence-based prevention, assessment, and intervention efforts.

Findings related to our second aim documented that there were few statistically significant sociodemographic differences between those with and without probable muscle dysmorphia. Only BMI was consistently different across those with and without probable muscle dysmorphia in both unadjusted and adjusted (i.e., logistic regression model adjusting for age, BMI, gender, race/ethnicity, sexual orientation, education, relationship status, and country) analyses. Specifically, those with a *lower* BMI had greater odds of experiencing probable muscle dysmorphia. This is an intriguing finding as BMI does not differentiate between fat mass and muscle mass [34] and, if just considering the prior research focused on bodybuilders, it might be presumed that those with muscle dysmorphia would have higher BMIs due to having greater muscle mass. However, considering the diagnostic criteria, which does not have a specific criterion related to weight nor BMI [1], and previous research [14, 35, 36], it may be that those with lower BMIs may be more likely to be preoccupied with a perceived lack of muscularity, resulting in greater muscle dissatisfaction, and a higher degree of muscularity-oriented behaviors. Indeed, the drive for muscularity is higher among individuals with lower BMIs [14]. Additionally, it has been described that there may be two distinct phenotypes of muscle dysmorphia: One that is focused specifically on being both muscular *and* lean, while the other specifically focused on being solely muscular [3]. It may be that those who fall within the muscular and lean phenotype are not as focused on building muscle mass (which would align more with the muscular phenotype), and the dual focus on muscularity *and* leanness results in a lower BMI (as opposed to the muscular phenotype likely equating to higher muscle mass, weight, and higher BMI). Given that the predominant body ideal for boys and men is one that is both muscular *and* lean [7], it may be that, given the findings from this study, this phenotype of muscle dysmorphia is more prevalent than the solely muscular phenotype. Certainly, future research is needed to continue to delineate nuanced presentations of muscle dysmorphia, including the specific sociodemographic factors that may be associated with these presentations.

Conversely, the lack of statistically significant differences across ages, genders, races/ethnicities, and sexual orientations is notable as it emphasizes that muscle dysmorphia occurs broadly across demographic groups, warranting a more universal approach to assessment, intervention, and prevention efforts. Specifically, healthcare professionals should be alerted to the lack of sociodemographic differences and ensure they are assessing for muscle dysmorphia across all ages, genders (particularly those whose natal sex is male), races/ethnicities, and sexual orientations. Prevention efforts should have targeted programming for all sociodemographic groups to ensure equity and inclusion.

Finally, findings related to our third aim underscore the strong association between probable muscle dysmorphia and standardized measures of muscle dysmorphia symptomatology and muscularity-oriented attitudes and behaviors. Specifically, those with probable muscle dysmorphia had significantly higher scores on the MDDI, including on the Drive for Size and Functional Impairment subscales, and Total score, as well as significantly higher scores on the DMS. Additionally, the vast majority of those with probable muscle dysmorphia (87.8%) scored above the previously established clinical cut-off score for the MDDI. Together, these findings provide validity to the operationalization previously outlined [13] that is used in this study and further underscore the severity of symptoms and impairment associated with muscle dysmorphia. Conversely, there were no significant differences between those with and without probable muscle dysmorphia and the Appearance Intolerance MDDI subscale. It may be that the current sample consists of boys and men who have a heightened level of body dissatisfaction, which aligns with prior research underscoring the high prevalence of weight change behaviors (i.e., weight gain and weight loss attempts and muscularity-oriented behaviors) [35–38] and body dissatisfaction [6] among boys and men. To a degree, these findings confirm the measurement of probable muscle dysmorphia in the current study and warrant future investigation on the validity and replicability of the measurement criteria used [13]. Increasing the validity of the measurement is particularly important to continue to investigate muscle dysmorphia in large-scale epidemiological research studies in the future.

There are limitations to this study that can be addressed in future research. The sample was recruited via non-probability sampling methods, which decreases confidence in the generalizability of the findings due to self-selection bias. However, the sample included broad representation across sociodemographic variables that were examined (e.g., age, gender, race/ethnicity, sexual orientation, education) and included participants across all 13 provinces and territories in Canada and all 10 zip code zones (i.e., major geographic regions) in the United States, underscoring the diversity of participants. Future research should replicate the findings using alternative sampling methods, particularly those that can generate a probability sample. Future research should also investigate the prevalence of muscle dysmorphia across geographic characteristics (e.g., rurality versus urbanicity). Additionally, the prevalence of probable muscle dysmorphia in the sample was similar to prior population-based research using similar measurement criteria [13], which provides some confidence in the representativeness of our sample. Future research is needed to assess probable muscle dysmorphia among nationally representative samples in Canada, the United States, and elsewhere to continue to build an understanding of the prevalence of muscle dysmorphia internationally. Furthermore, only two social media platforms were used to recruit participants. However, it should be noted that smartphone access and use of social media, specifically Instagram and Snapchat, is highly prevalent among boys and men ages 15–35 [39–43], resulting in access to a large sampling frame. The measurement of probable muscle dysmorphia was not conducted via a clinical interview, which may result in an inaccurate estimate of the prevalence of muscle dysmorphia. However, the measurement was based on operationalized clinical criteria and utilized a mix of standardized measures and researcher-designed items, increasing reliability, and thus, may represent a resource and cost-effective approach for epidemiological studies. Future research is needed to provide further empirical support for the criteria set forth by Mitchison and colleagues (2021) utilizing clinical samples, which will aid future epidemiological and clinical research on muscle dysmorphia. Lastly, there was a pattern of missing data not completely at random, which should be considered when interpreting the findings. Strengths of the study include the international sample, demographic diversity, and diagnostic criteria-based measurement of muscle dysmorphia.

## Conclusion

The findings from this study underscore the relatively high prevalence of probable muscle dysmorphia in an international sample of boys and men. Importantly, probable muscle dysmorphia presented across sociodemographic identifiers, including ages, genders, races/ethnicities, and sexual orientations, highlighting the broad occurrence of this condition. Findings continue to describe muscle dysmorphia that can be utilized for future investigation, prevention, assessment, and intervention efforts.

## Electronic supplementary material

Below is the link to the electronic supplementary material.

Supplementary Material 1

## Data Availability

No datasets were generated or analysed during the current study.
