# Supplementary material for: Prevalence and correlates of muscle dysmorphia in a sample of boys and men in Canada and the United States
Source: J Eat Disord. 2025 Mar 17;13:47. doi: 10.1186/s40337-025-01233-x (PMC11916914; doi:10.1186/s40337-025-01233-x)
Supplement: Supplementary file 1 — Supplementary Material 1 [file 40337_2025_1233_MOESM1_ESM.docx]

| Supplementary Table 1.  Muscle dysmorphia diagnostic criteria^1^ and operationalization in the current study^2^ | | |
| --- | --- | --- |
| Criteria | Operationalization | α^5^ |
| A. The person has a preoccupation with the idea that one’s body is not sufficiently lean and muscular. Characteristic associated behaviors include long hours of lifting weights and excessive attention to diet | ≥ 1.5 SD above mean on the DFS subscale of the MDDI^3^  To enhance the validity of Mitchison et al.'s (2021) original operationalization, the current study defined a participant's preoccupation with muscularity as having a score of ≥ 1.5 standard deviations above the mean on the Drive for Size subscale of the Muscle Dysmorphic Disorder Inventory (Hildebrandt et al., 2004). | MDDI DFS subscale: 0.86 |
| B. The preoccupation causes clinically significant distress or impairment in social, occupational, or other important areas of functioning, as demonstrated by at least two of the following four criteria: | At least 2 of the following: |  |
| (a) The individual frequently gives up important social, occupational, or recreational activities because of a compulsive need to maintain his or her workout and diet schedule; | A score of ≥4 (indicating often to always) on one or more of the following DMS/MDDI items:  1. “I think that my weight-training schedule interferes with other aspects of my life” 2. “I pass up social activities with friends because of my workout schedule” 3. “I pass up chances to meet new people because of my workout schedule” |  |
| (b) The individual avoids situations where his or her body is exposed to others, or endures such situations only with marked distress or intense anxiety; | A score of ≥ 4 (indicating moderately to markedly) on the EDE-Q item:  “Over the past 4 weeks (28 days), how uncomfortable have you felt about others seeing your shape or figure (for example, in communal changing rooms, when swimming, or wearing tight clothes)?” |  |
| (c) The preoccupation about the inadequacy of body size or musculature causes clinically significant distress or impairment in social, occupational, or other important areas of functioning; | PedsQL physical or psychosocial score > 1 SD below the sample mean (indicating significant impairment)  OR  K10 total score ≥ 15 (indicating mild to severe distress) | PedsQL Adolescent: 0.86  PedsQL Young Adult: 0.88  K10: 0.92 |
| (d) The individual continues to work out, diet, or use ergogenic (performance enhancing) substances despite knowledge of adverse physical or psychological consequences | Score ≥ 4 (indicating often to always) on the DMS item: “I lift weights to build up muscle”  OR  Score ≥ 12 days on the researcher-developed item: “In the past 28 days, how many days have you been on a very high-protein diet as a means of controlling your shape or weight?”  OR  Score ≥ 1 on the researcher-developed item: “In the past 28 days, how many times have you taken anabolic steroids as a means of increasing your muscularity?” |  |
| C. The primary focus of the preoccupation and behaviors is on being too small or inadequately muscular, as distinguished from fear of being fat, as in anorexia nervosa, or a primary preoccupation only with other aspects of appearance, as in other forms of BDD | Score ≥ 4 (indicating often to always) on the DMS item: “I wish that I were more muscular”  AND  Exclusion if meeting criteria for anorexia nervosa, bulimia nervosa, atypical anorexia nervosa, or subthreshold bulimia nervosa^4^ |  |
| SD = Standard deviation; DFS = Drive for Size; MDDI = Muscle Dysmorphic Disorder Inventory; DMS = Drive for Muscularity Scale; EDE-Q = Eating Disorder Examination Questionnaire; PedsQL = Pediatric Quality of Life Inventory; K10 = Kessler 10; BDD = Body dysmorphic disorder  ^1^ Based on Pope, H. G., Gruber, A. J., Choi, P., Olivardia, R., & Phillips, K. A. (1997). Muscle dysmorphia: An underrecognized form of body dysmorphic disorder. *Psychosomatics*, *38*(6), 548–557. <https://doi.org/10.1016/S0033-3182(97)71400-2>  ^2^ Adapted from Mitchison, D., Mond, J., Griffiths, S., Hay, P., Nagata, J. M., Bussey, K., … Murray, S. B. (2022). Prevalence of muscle dysmorphia in adolescents: findings from the EveryBODY study. *Psychological Medicine*, *52*(14), 3142–3149. doi:10.1017/S0033291720005206  ^3^ Only minor change to Mitchison et al.’s operationalization.  ^4^ For eating disorder criteria, see Mitchison, D., Mond, J., Bussey, K., Griffiths, S., Trompeter, N., Lonergan, A., … Hay, P. (2020). DSM-5 full syndrome, other specified, and unspecified eating disorders in Australian adolescents: prevalence and clinical significance. *Psychological Medicine*, *50*(6), 981–990. doi:10.1017/S0033291719000898  ^5^ Internal consistency using Cronbach’s alphas of all full measures included in the operationalization among participants from The Study of Boys and Men (N = 1,553). | | |

| Supplementary Table 2.  Demographic Survey Questions | | | | |
| --- | --- | --- | --- | --- |
| Variable | Question | Original Survey Response Options | Recoded Categories | |
| Sex at birth | What sex were you assigned at birth on your original birth certificate? | Male Female Intersex Prefer not to say | 0=Female 1=Male  Note: There were no observations for “intersex” and “prefer not to say”. | |
| Gender identity | What is your current gender identity? Select all that apply. | Boy/man Girl/woman Trans male/Trans man Trans female/Trans woman Genderqueer/Gender non-conforming Gender non-binary  Self-identify (please specify)  Prefer not to say | 0=Boy/Man 1=Trans Man 2=Gender Expansive & Other  Note: “Prefer not to say” was coded as missing. | |
| Race/ethnicity | In our society, people are often described by their race/ethnicity or racial/ethnic background.   Which racial/ethnic category(ies) best describes you? Check all that apply. | Black (ex. African, African Canadian/American, Afro-Caribbean descent) East Asian (ex. Chinese, Japanese, Korean, Taiwanese descent) Indigenous (ex. First Nations, Inuk/Inuit, Métis descent) Latin American (ex. Hispanic or Latin American descent) Middle Eastern (ex. Arab, Persian, West Asian descent (e.g., Afghan, Egyptian, Iranian, Kurdish, Lebanese, Turkish)) South Asian (ex. South Asian descent (e.g., Bangladeshi, Indian, Indo-Caribbean, Pakistani, Sri Lankan)) Southeast Asian (ex. Cambodian, Filipino, Indonesian, Thai, Vietnamese, or other Southeast Asian descent) White (European descent [e.g., British, French, Italian, Portuguese, Ukrainian, Russian]))  Another race category (please specify) Do not know Prefer not to answer | 0=White 1=Black 2=Asian (East Asian, South Asian, Southeast Asian) 3=Latin American 4=Other (Middle Eastern, Indigenous, Other) 5=Multi-Racial  Note: “Prefer not to answer” and “Don’t know” were coded as missing. | |
| Sexual orientation | What is your current sexual orientation? Select all that apply. | Asexual Bisexual Gay/Lesbian Heterosexual ("straight") Pansexual Queer Questioning Other (please specify) Prefer not to say | 0=Heterosexual 1=Gay/Lesbian 2=Bisexual  3=Queer 4=Questioning, Other, Asexual, Pansexual, Other   Note: “Prefer Not to Say” were coded as missing. | |
| Relationship status | What is your current relationship status? | Single Dating (not living together) Dating (living together) Civil union/domestic partnership Married Other (please specify) | 0=Single 1=In Relationship  Note: “Other” responses were recategorized accordingly. | |
| Highest completed education | What is the highest level of formal education that you have completed? | 8th Grade or lower 9th Grade 10th Grade 11th Grade 12th Grade/high school diploma or equivalent College diploma or trade school certificate University degree (e.g., BS, BA) Master's degree (e.g., MSc, MSW, MPH) Doctoral degree (e.g., PhD, MD, JD) Other (please specify) | 0=HS or less 1=College or undergrad degree 2=Master’s degree or higher  Note: “Other” responses were recategorized into their respective group. | |
| Location | What is your postal code or zip code?  Please use the following formats: "M5S 1V4" (postal code); "10001" (zip code) | Open response | 0=Canada  1=United States |  |

| Supplementary Table 3.  Patterns of Missing Data | | | | |
| --- | --- | --- | --- | --- |
|  | Included  (n = 1,488) | Excluded  (n = 65) |  |  |
|  | % | % | *p*^a^ | *t / V*^b^ |
| Age (*M* [SD]) | 24.1 (5.6) | 23.6 (6.2) | .512 | 0.65 |
| BMI (*M* [SD]) | 25.2 (6.2) | 27.4 (6.7) | .015 | -2.42 |
| Gender |  |  | .085 | 0.06 |
| Cisgender Boy/Man | 82.4 | 72.4 |  |  |
| Trans Boy/Man | 7.4 | 13.8 |  |  |
| Gender Expansive and Other | 10.2 | 13.8 |  |  |
| Race/Ethnicity |  |  | .408 | 0.06 |
| White | 65.3 | 75.4 |  |  |
| Black | 3.1 | 3.5 |  |  |
| Asian | 11.0 | 5.3 |  |  |
| Latin American | 4.0 | 5.3 |  |  |
| Other | 3.6 | 0.0 |  |  |
| Multi-Racial | 13.0 | 10.5 |  |  |
| Sexual Orientation |  |  | .363 | 0.05 |
| Heterosexual | 48.2 | 44.9 |  |  |
| Gay | 19.9 | 13.8 |  |  |
| Bisexual | 13.9 | 13.8 |  |  |
| Queer | 7.9 | 10.3 |  |  |
| Questioning and Other | 10.1 | 17.2 |  |  |
| Education |  |  | .014 | 0.08 |
| High School Diploma or Less | 43.8 | 52.7 |  |  |
| College or Undergraduate Degree | 39.7 | 45.5 |  |  |
| Master’s Degree or Higher | 16.5 | 1.8 |  |  |
| Relationship Status |  |  | .062 | -0.05 |
| Single | 54.8 | 67.2 |  |  |
| In a Relationship | 45.2 | 32.8 |  |  |
| Country |  |  | .478 | 0.02 |
| Canada | 58.4 | 53.2 |  |  |
| United States | 41.6 | 46.8 |  |  |
| Participants were excluded due to missing data for any item in the probable muscle dysmorphia algorithm.  ^a^ Statistical significance was determined using chi-square tests for categorial variables and independent samples *t*-tests for continuous variables.  ^b^ *t* statistic from independent samples *t*-tests for continuous variables and Cramér’s V for categorical variables. | | | | |
